# International standards for symphysis-fundal height based on serial measurements from the Fetal Growth Longitudinal Study of the INTERGROWTH-21^st^ Project: prospective cohort study in eight countries

**DOI:** 10.1136/bmj.i5662

**Published:** 2016-11-07

**Authors:** Aris T Papageorghiou, Eric O Ohuma, Michael G Gravett, Jane Hirst, Mariangela F da Silveira, Ann Lambert, Maria Carvalho, Yasmin A Jaffer, Douglas G Altman, Julia A Noble, Enrico Bertino, Manorama Purwar, Ruyan Pang, Leila Cheikh Ismail, Cesar Victora, Zulfiqar A Bhutta, Stephen H Kennedy, José Villar

**Affiliations:** 1 Nuffield Department of Obstetrics & Gynaecology and Oxford Maternal & Perinatal Health Institute, Green Templeton College, University of Oxford, Oxford, UK; 2 Centre for Statistics in Medicine, Nuffield Department of Orthopaedics, Rheumatology & Musculoskeletal Sciences, University of Oxford, Oxford, UK; 3 Center for Perinatal Studies, Swedish Medical Center, Seattle, WA, USA; 4 Global Alliance to Prevent Prematurity and Stillbirth, Seattle, WA, USA; 5 Departamento Materno-Infantil, Universidade Federal de Pelotas, Pelotas, RS, Brazil; 6 Programa de Pós-Graduação em Epidemiologia, Universidade Federal de Pelotas, Pelotas, RS, Brazil; 7 Faculty of Health Sciences, Aga Khan University, Nairobi, Kenya; 8 Department of Family & Community Health, Ministry of Health, Muscat, Sultanate of Oman; 9 Department of Engineering Science, University of Oxford, Oxford, UK; 10 Dipartimento di Scienze Pediatriche e dell’Adolescenza, Cattedra di Neonatologia, Università degli Studi di Torino, Turin, Italy; 11 Nagpur INTERGROWTH-21st Research Centre, Ketkar Hospital, Nagpur, India; 12 School of Public Health, Peking University, Beijing, China; 13 Division of Women & Child Health, Aga Khan University, Karachi, Pakistan

## Abstract

**Objective** To create international symphysis-fundal height standards derived from pregnancies of healthy women with good maternal and perinatal outcomes.

**Design** Prospective longitudinal observational study.

**Setting** Eight geographically diverse urban regions in Brazil, China, India, Italy, Kenya, Oman, United Kingdom, and United States.

**Participants** Healthy, well nourished pregnant women enrolled into the Fetal Growth Longitudinal Study component of the INTERGROWTH-21^st^ Project at 9-14 weeks’ gestation, and followed up until birth.

**Main outcome measures** Symphysis-fundal height was measured every five weeks from 14 weeks’ gestation until birth using standardised methods and dedicated research staff who were blinded to the symphysis-fundal height measurements by turning the tape measure so that numbers were not visible during examination. The best fitting curve was selected using second degree fractional polynomials and further modelled in a multilevel framework to account for the longitudinal design of the study.

**Results** Of 13 108 women screened in the first trimester, 4607 (35.1%) met the study entry criteria. Of the eligible women, 4321 (93.8%) had pregnancies without major complications and delivered live singletons without congenital malformations. The median number of symphysis-fundal height measurements was 5.0 (range 1-7); 3976 (92.0%) women had four or more measurements. Symphysis-fundal height measurements increased almost linearly with gestational age; data were used to determine fitted 3rd, 50th, and 97th centile curves, which showed excellent agreement with observed values.

**Conclusions** This study presents international standards to measure symphysis-fundal height as a first level screening tool for fetal growth disturbances.

## Introduction

Assessment of fetal growth is one of the aims of antenatal care, in order to identify small and large for gestational age fetuses at increased risk of perinatal morbidity and mortality. In low risk pregnancies, serial measurement of symphysis-fundal height (SFH) is recommended as a simple, inexpensive, first level, screening tool.^1^ ^2^ ^3^ Fetal growth in high risk pregnancies should be monitored with serial ultrasound scans by plotting anthropometric measures against international standards.^4^ In low and middle income countries, where access to ultrasound machines and trained ultrasonographers is limited, SFH measurement is often the screening tool of choice for fetal growth disturbances.

The sensitivity of SFH measurement to detect small for gestational age fetuses has been assessed in three systematic reviews.^5^ ^6^ ^7^ Observational cohort studies show wide ranges of sensitivity from 17% to 93%. There is also marked study heterogeneity mainly due to the variety of methods used, including varying thresholds for defining small for gestational age and the use of multiple SFH charts.^5^ In fact, we believe that at least 21 different, locally derived SFH charts are currently being used in clinical practice worldwide.^8^ ^9^ ^10^ ^11^ ^12^ ^13^ ^14^ ^15^ ^16^ ^17^ ^18^ ^19^ ^20^ ^21^ ^22^ ^23^ ^24^ ^25^ ^26^ ^27^ ^28^ ^29^

To improve the care offered to women worldwide, we have developed international SFH standards, which were derived from the same eight urban populations of healthy, well nourished women in the INTERGROWTH-21^st^ Project. The project has already generated international standards for ultrasound dating of early pregnancy^30^ and assessment of fetal growth,^4^ maternal weight gain,^31^ newborn size for gestational age and sex,^32^ ^33^ and preterm postnatal growth.^34^

## Methods

INTERGROWTH-21^st ^was a multicentre, multiethnic, population based project, conducted between 2009 and 2014 in eight countries.^35^ The project’s primary aim was to study growth, health, nutrition, and neurodevelopment from less than 14 weeks’ gestation to 2 years of age, using the same conceptual framework as the World Health Organization Multicentre Growth Reference Study.^36^

The details of population selection have been described elsewhere.^35^ ^37^ In brief, all institutions providing obstetric care in eight urban areas in Brazil, China, India, Italy, Kenya, Oman, UK, and USA, with no or low levels of major, known, non-microbiological contamination, were chosen as study sites. From these populations, healthy women with a naturally conceived singleton pregnancy, and who met the individual inclusion criteria, were prospectively recruited into the Fetal Growth Longitudinal Study, one of the main components of the INTERGROWTH-21^st^ Project.

Gestational age was estimated on the basis of the last menstrual period provided that the date was certain, the woman had a regular 24-32 day menstrual cycle, she was not using hormonal contraception or breastfeeding in the preceding two months, and the estimated gestational age (based on the last menstrual period) agreed (within seven days) with a standardised measurement of fetal crown rump length at 9^+0^ to 13^+6^ weeks’ gestation.^30^ ^38^ ^39^ Follow-up visits occurred every five weeks (within one week either side)—that is, possible ranges were 14-18, 19-23, 24-28, 29-33, 34-38, and 39-42 weeks’ gestation.

At each visit, dedicated research staff who had undergone rigorous training and standardisation used the same protocols at all sites. Staff measured SFH first before taking fetal ultrasound measurements. With the woman in the supine position, having emptied her bladder, SFH was measured with a non-elastic metric tape (Chasmors) provided to all sites. After the start of the tape was positioned with one hand over the upper border of the symphysis pubis bone, the tape was placed in a straight line over the uterus until loss of resistance was felt when reaching the fundus. With the cubital edge of the hand used to sustain the tape in place at the point of the fundus, the tape was turned so that the numbers were visible to record the value to the nearest complete half centimetre.^35^

The process was then repeated to obtain a second measurement. Although it was not possible to blind the research staff to the gestational age at each visit, all SFH measurements were taken in a blinded fashion to reduce expected value bias by turning the tape measure so that no numbers were visible during the examination.

According to prespecified criteria, we excluded pregnancies complicated by fetal death or congenital abnormality, catastrophic or severe medical conditions (such as cancer or HIV); those with severe unanticipated conditions related to pregnancy that needed admission to hospital (such as eclampsia or severe pre-eclampsia); and those identified during the study who no longer fulfilled the entry criteria (such as women who started smoking during pregnancy or had an episode of malaria).

### Statistical analysis

Sample size considerations are reported elsewhere.^4^ In keeping with the overall analysis strategy, our aim was to produce centiles that change smoothly with gestational age and maximise simplicity without compromising model fit.

The best fitting powers for the median SFH were provided by second degree fractional polynomials and further modelled in a multilevel framework to account for the longitudinal study design. To obtain an equation for the standard deviation, we modelled the resulting variance components from the multilevel model that accounts for the correlations between and within participants using fractional polynomials. Goodness of fit was based on visual inspection of the overall model fit by comparing quantile-quantile (q-q) plots of the residuals, and the distribution of fitted Z scores across gestational ages.

Tables containing medians and standard deviations of the SFH by gestational age, expressed in completed weeks of gestation (as recommended by WHO International Statistical Classification of Diseases and Related Health Problems, 10th revision), as well as printable charts, are freely available on the INTERGROWTH-21^st^ website (https://intergrowth21.tghn.org; supplementary appendix).

For the analysis of estimation of gestational age as a function of SFH only, the problem of data truncation at the lower and upper gestational ages was overcome by use of the same approach applied to crown rump length data in the Fetal Growth Longitudinal Study.^40^ By use of the equation for SFH size according to gestational age (size chart), 40 observations for each day were simulated between 10^+0^ and 13^+6^ weeks’ gestation (n=1120). Similarly, 40 observations for each day were simulated between 40^+0^ and 46^+0^ weeks’ gestation (n=1720)—that is, about the same number of observations for each day of gestational age in the untruncated dataset. After simulation, we restricted the data based on SFH by excluding actual and simulated values below 12 cm or above 38 cm (because gestational age assessment based on SFH is not undertaken clinically beyond these limits) and visually inspecting a plot of the data to assess that the truncation problem had been overcome. This process resulted in a total of 814 simulated data being included in the final dataset. Using the augmented dataset, we conducted fractional polynomial regression analyses using the xrigls function in Stata (version 11.2) to model the mean and standard deviation for each biometric variable.^40^

### Patient involvement

The steering committee of the project included a female lay adviser who had previously chaired an ethics committee; she contributed to the study design including developing plans for participant recruitment and advice about the ethical conduct of the research.^41^

## Results

Of 13 108 women screened in the first trimester, 4607 women met the study entry criteria and were recruited across the eight study sites; their characteristics have been reported in detail previously.^42^ Of these women, 4321 (93.8%) delivered live singletons without congenital malformations (fig 1), but 82 were excluded from the analysis because all SFH measures were missing (n=81) or a single measure was above 38 cm (n=1). In addition, we excluded 59 outlier observations (but not any women as a result), defined as SFH values greater than five standard deviations above the mean. Therefore, for the analysis of SFH for gestational age, 20 566 measurements from 4239 women were used, which contained no simulated data.

**Figure f1:**
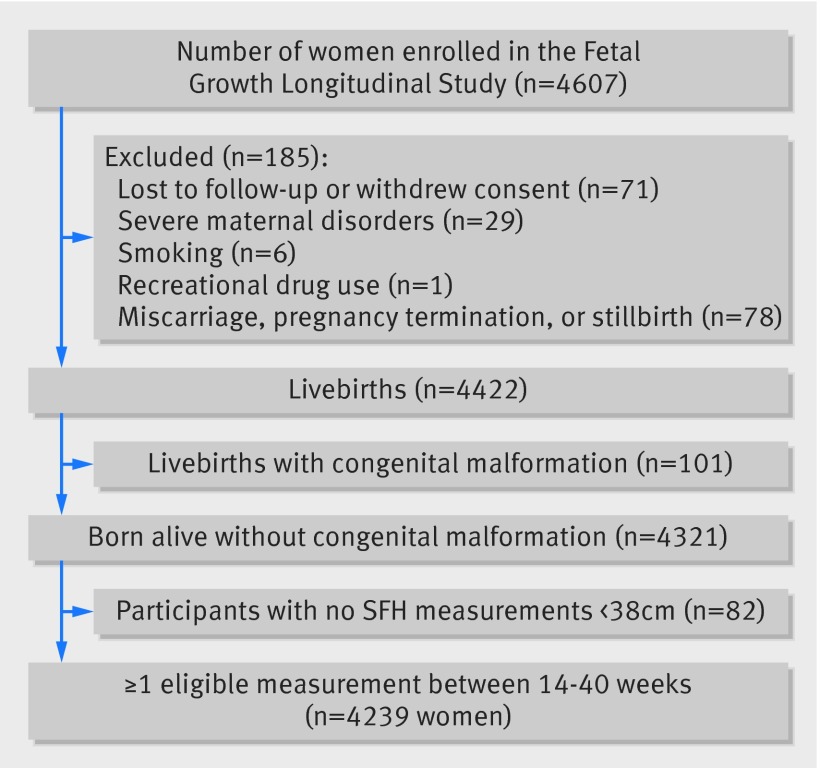
**Fig 1** Flowchart of recruitment of participants through the study. SFH=symphysis-fundal height

For the estimation of gestational age from SFH, the dataset was augmented with simulated observations: 1120 at the lower end and 1720 at the upper end. Subsequently, we excluded real and simulated SFH data below 12 cm (n=604 observations) and above 38 cm (n=1842 observations) to obtain the final analysis sample of 20 837 observations. As a result, in the analysis of gestational age for SFH, 814 were simulated and 20 023 were measured data points in 4239 women (fig 1). Baseline characteristics and perinatal events of the study population (n=4239, excluding simulated observations) are shown in table 1. The median number of SFH measurements in all women was 5.0 (range 1-7); 3976 (92.0%) women had four or more measurements. Of 20 907 measurements, 18 061 (86.4%) were taken within the stipulated gestational age window, confirming strict adherence to the study protocol. As the relation between SFH and gestational age was almost linear, complex methods that allow for skewness and kurtosis were not required.

**Table 1 tbl1:** Baseline characteristics and perinatal events of study population (n=4239)

Characteristic	Mean (standard deviation)	No (%) of women
Maternal age (years)	28.4 (3.9)	—
Maternal height (cm)	162.2 (5.8)	—
Maternal weight (kg)	61.3 (9.1)	—
Body mass index	23.3 (3.0)	—
Gestational age at first visit (weeks)	11.8 (1.4)	—
Nulliparous	—	2907 (68.6)
Pre-eclampsia	—	31 (<1)
Preterm birth (<37 weeks’ gestation)	—	192 (4.5)
Term LBW <2500 g (≥37 weeks’ gestation)	—	127 (3)
Male infant	—	2106 (49.7)
Birth weight (≥37 weeks’ gestation; kg)	3.267 (0.444)	—

Maternal baseline characteristics were measured at less than 14 weeks’ gestation. LBW=low birth weight.

The relation between SFH and gestational age was best expressed by the equation: median SFH=5.133374 + 0.1058353119 × (GA^2^) − 0.0231295 × (GA^2^) × ln (GA), 

where GA is gestational age. The standard deviation of the SFH was expressed by: 

SD (SFH) = 0.9922667 + 0.0258087 × GA 

(where SD is standard deviation, SFH is in cm, GA is in exact weeks in decimals (eg, 36^+0^ and 36^+1^ weeks’ gestation are 36.0 and 36.14 weeks’ gestation, respectively), and ln is the natural logarithm).

Assessment of the goodness of fit—by comparing smoothed 3rd, 50th, and 97th centile curves and observed values—showed excellent agreement (fig 2). Overall, the mean differences between smoothed and observed centiles for the 3rd, 50th, and 97th centiles, were small: 0.28 cm (standard deviation 0.61), 0.04 cm (0.37), and 0.45 cm (0.51), respectively.

**Figure f2:**
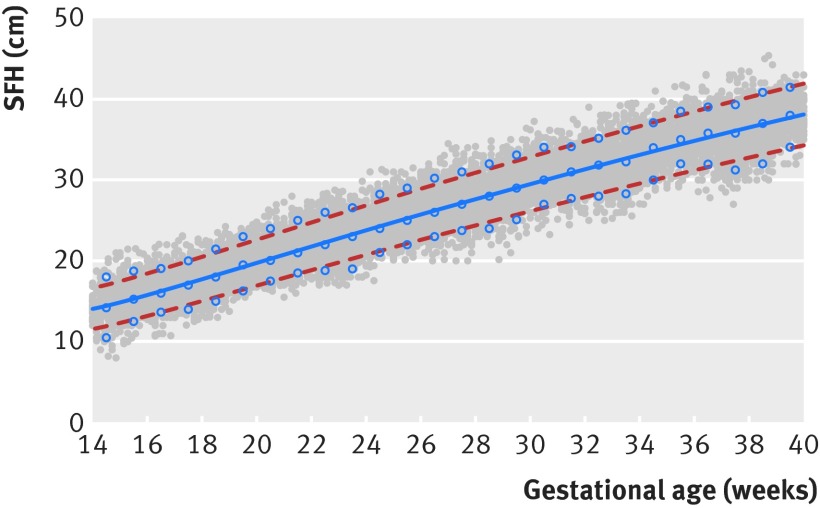
**Fig 2** Scatterplot showing symphysis-fundal height (SFH) with gestational age. Lines=fitted smoothed 3rd (bottom dashed line), 50th (middle solid line), and 97th (top dashed line) centile curves; open blue circles=empirical values for each week of gestation; grey circles=actual observations

The equations for the median and standard deviation from the regression models allow calculations of any desired centiles according to gestational age in exact weeks. For example, centiles can be calculated as the mean plus or minus Z×standard deviation, where Z is −1.88, −1.645, −1.28, 0, 1.28, 1.645, and 1.88 for the 3rd, 5th, 10th, 50th, 90th, 95th, and 97th centiles, respectively. A computerised application for the use of these data for individuals and datasets will be made available (https://intergrowth21.tghn.org).

Table 2 and figure 3 present the international SFH standards for clinical use, and there is a printable chart available (supplementary appendix; can also be found at https://intergrowth21.tghn.org/ under “INTERGROWTH Standards & Tools”).

**Table 2 tbl2:** International symphysis-fundal height standards for clinical use, rounded to the nearest 0.5 cm

Gestational age (exact weeks*)	No of SFH observations	Centiles of SFH (cm)						
		3rd	5th	10th	50th	90th	95th	97th
16	965	13.0	13.5	14.0	16.0	17.5	18.0	18.5
17	1140	14.0	14.5	15.0	17.0	18.5	19.0	19.5
18	872	15.0	15.5	16.0	18.0	19.5	20.0	20.5
19	508	16.0	16.5	17.0	19.0	20.5	21.0	21.5
20	747	17.0	17.5	18.0	20.0	21.5	22.0	22.5
21	872	18.0	18.0	19.0	21.0	22.5	23.5	23.5
22	991	19.0	19.0	20.0	22.0	24.0	24.5	24.5
23	960	20.0	20.0	20.5	23.0	25.0	25.5	25.5
24	618	20.5	21.0	21.5	24.0	26.0	26.5	27.0
25	702	21.5	22.0	22.5	24.5	27.0	27.5	28.0
26	836	22.5	23.0	23.5	25.5	28.0	28.5	29.0
27	934	23.5	24.0	24.5	26.5	29.0	29.5	30.0
28	939	24.5	25.0	25.5	27.5	30.0	30.5	31.0
29	724	25.5	26.0	26.5	28.5	31.0	31.5	32.0
30	744	26.5	26.5	27.5	29.5	32.0	32.5	33.0
31	772	27.0	27.5	28.0	30.5	33.0	33.5	34.0
32	927	28.0	28.5	29.0	31.5	34.0	34.5	35.0
33	964	29.0	29.5	30.0	32.5	34.5	35.5	36.0
34	747	29.5	30.0	31.0	33.0	35.5	36.5	36.5
35	760	30.5	31.0	31.5	34.0	36.5	37.0	37.5
36	714	31.5	31.5	32.5	35.0	37.5	38.0	38.5
37	1119	32.0	32.5	33.0	35.5	38.0	39.0	39.5
38	603	33.0	33.0	34.0	36.5	39.0	39.5	40.0
39	337	33.5	34.0	34.5	37.0	40.0	40.5	41.0
40	120	34.0	34.5	35.5	38.0	40.5	41.5	42.0

*That is, week plus 0 days.

**Figure f3:**
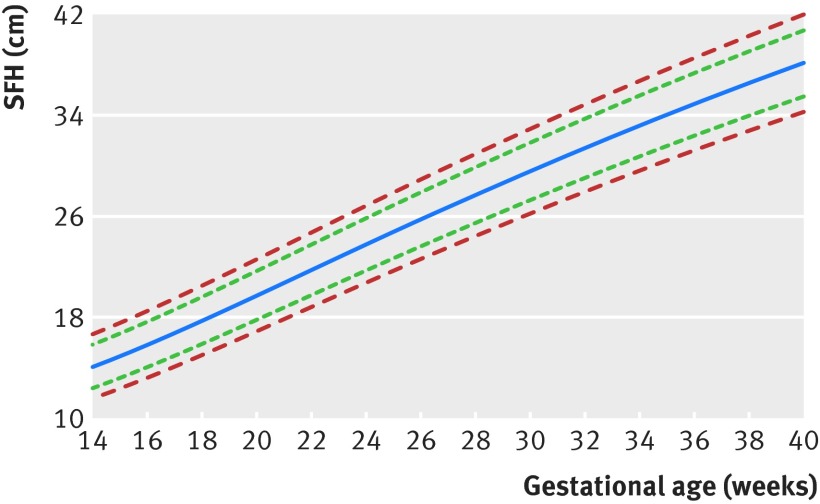
**Fig 3** International symphysis-fundal height (SFH) standards for clinical use. Lines (from bottom to top)=3rd, 10th, 50th, 90th, and 97th centiles. A printable chart is available in the supplementary appendix (can also be found at https://intergrowth21.tghn.org/ under “INTERGROWTH Standards & Tools”)

Estimation of gestational age from SFH was expressed by the equation: GA (exact weeks) = 6.585838 − 2.7072585 × (SFH^0.5^) + 1.295291 × (SFH), where GA is gestational age. Assessment of the goodness of fit showed excellent agreement; the equation can be used to estimate gestational age in settings where more reliable information, such as ultrasound dating of the pregnancy, is not available (fig 4).

**Figure f4:**
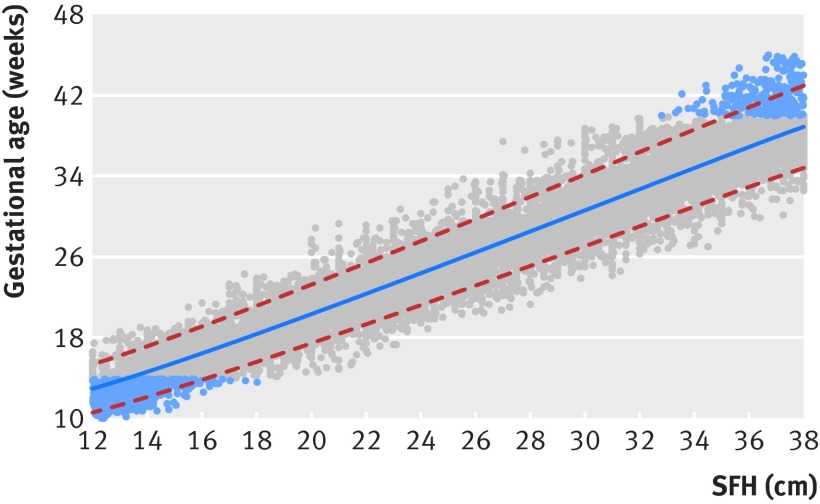
**Fig 4** Gestational age as a function of symphysis-fundal height (SFH). Lines=10th (bottom dashed line), 50th (middle solid line), and 90th (top dashed line) centiles; grey circles=actual observations; blue circles=simulated data to overcome truncation (see text for details)

It is clear that SFH, as an indirect measure of the growth of the fetus and uterine volume, has limitations. Analysis of the duplicate SFH measurements obtained from all women showed that the 95% limits of agreement were about 1.5 cm (fig 5). Previous studies have demonstrated that interobserver agreement exceeds this value significantly, with 95% limits of agreement of around 6 cm.^43^

**Figure f5:**
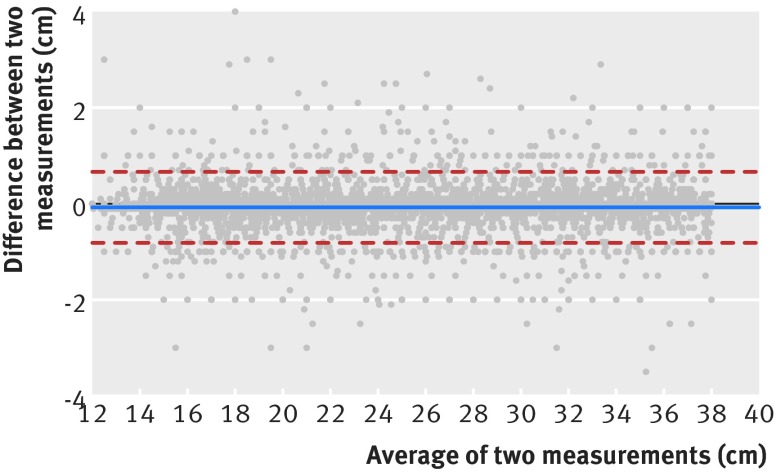
**Fig 5** Bland Altman plot of intraobserver variation of symphysis-fundal height (SFH) measurement; the central line shows the mean difference between observation 1 and 2 (solid line, 0.07 cm) while the dashed lines show the 95% limits of agreement (upper dashed line 0.66cm, lower dashed line 0.81 cm)

All the clinical tools arising from the INTERGROWTH-21^st^ Project, including the SFH charts, are freely available on our website for healthcare professionals and patients to use.

## Discussion

### Principal findings

Based on the recommendations of the 1995 WHO Expert Committee regarding “the use and interpretation of anthropometry,”^44^ we selected a population based cohort of healthy, well nourished mothers at low risk of adverse outcomes to generate international standards describing optimal maternal, fetal, and newborn parameters. The SFH standards presented here are the fifth in the series to use the same conceptual approach and dataset derived from the mothers and babies who participated in the INTERGROWTH-21^st^ Project.

### Strengths and limitations of study

Although SFH measurement is a simple, inexpensive, first level screening tool in both high and low income settings, it is associated with a wide range of sensitivities for detecting small for gestational age, principally because of the different measurement methods, charts, and thresholds used to perform an ultrasound scan. Another limitation of existing reference charts is that they use uncorroborated menstrual dates, which can cause artificial flattening of the growth curve at term, probably due to errors in dating that overestimate the length of gestation. The strict strategy for dating pregnancies based on the last menstrual period, corroborated by first trimester ultrasound in all women taking part in the INTERGROWTH-21^st^ Project, shows no such flattening at term in the fetal growth standards.^4^

The standards we have developed overcome many of these methodological limitations. Their widespread implementation will allow clinical practice to be unified and comparisons to be made across international studies using a single chart derived from the same low risk population as the other INTERGROWTH-21^st^ standards. One possible limitation of our study is that the measurements were obtained under near optimal conditions—all the dedicated staff taking the measurements were well trained, used the same protocol under blinded conditions, and had no time restrictions to complete the examinations. Consequently, the intraobserver variability in the measurements was probably better than that reported previously. Under less than ideal conditions, the measures obtained might also have been different. However, our actual aim was to report measures under optimal conditions so that they can serve as a standard.

These international, prescriptive standards, which describe optimal growth, have the potential to improve pregnancy outcomes by reducing the risk of failure to recognise a fetus of small for gestational age through the use of locally produced reference charts that include data derived from high risk mothers.

### Comparison with other studies

The introduction of these international standards should be relatively simple because the practice of SFH measurement is so well established in both developed and developing countries. In the UK, for example, the National Institute for Health and Care Excellence^45^ recommends serial measurement of SFH at each antenatal appointment from 24 weeks’ gestation. In a 2011 systematic review, Imdad and colleagues^6^ concluded that detection and management of intrauterine growth restriction (IUGR) using maternal body mass index screening, SFH measurement, and targeted ultrasound followed by appropriate management could be an effective method of reducing IUGR related stillbirths. This finding remains to be proven in a clinical trial. In general, referral from first level SFH screening to more detailed assessment is warranted if SFH measures are below or above a certain centile, provided that the estimation of gestational age is accurate, or if repeat measures show a drop or acceleration across centiles. We have made such implementation easier by posting freely available, printable SFH charts on our website for plotting the measures.

It would not be appropriate for us, however, to make recommendations about the cutoff values that warrant referral. In general, first level screening tests for anthropometric measures use a relatively high screen positive rate (usually cutoff values at the 10th and 90th centiles) in order to maximise sensitivity. Considering that the action taken for false positives is non-invasive (that is, closer surveillance), this might be acceptable. However, in service deprived areas with high rates of IUGR, such cutoff values could produce an unmanageable number of referrals and should be adapted.

What we can say is that the use of international, prescriptive standards, which describe optimal growth, instead of a wide range of locally derived reference charts, should reduce the risk of failing to diagnose both restricted and excessive fetal growth and will facilitate comparisons across populations.

### Conclusions and policy implications

Assessment of fetal size and growth by SFH measurement is a simple and inexpensive clinical activity, widely used during antenatal care in both high and low income settings. The international standards we present will go some way to reducing the wide range in sensitivity for the detection of small for gestational age. Given that SFH measurement constitutes a first level screen, with suspected pregnancies referred—without treatment—for further non-invasive investigations with ultrasound, a high false positive rate could be acceptable if it leads to improved detection rates overall. Future work should concentrate on the optimal frequency of SFH measurement to maximise detection.

Assessment of fetal growth using SFH remains an important first level screening tool during routine antenatal care. We recommend the use of the new international SFH standards in combination with standardised measurement methodology to unify and improve clinical practice. Plotting measurements in the medical records with these tools should be undertaken to identify women who require referral for an ultrasound scan.

**What is already known on this topic**

Symphysis-fundal height (SFH) measurement is routinely performed during antenatal care to screen for fetal growth disturbances
Three systematic reviews have assessed the sensitivity of SFH measurement to detect small for gestational age; observational cohort studies show wide ranges of sensitivity from 17% to 93%
It is likely that at least part of the reason for this wide range of sensitivities is that the methodology has never been standardised and a variety of different, locally derived SFH charts are currently in use

**What this study adds**

This international study uses a prescriptive approach to develop international SFH standards using the eight urban populations of healthy, well nourished women in the INTERGROWTH-21^st^ Project
These scientifically robust standards should be used as a first level screening tool to alert clinicians to disturbances in fetal growth, which should initiate referral for detailed assessment with ultrasound, if the resource is available
The charts complement unified tools for pregnancy assessment, including international standards for ultrasound dating of early pregnancy and assessment of fetal growth, maternal weight gain, newborn size for gestational age/sex, and preterm postnatal growth

## Web Extra

Extra material supplied by the author

**Supplementary appendix:** International Standards for Symphysis-Fundal Height

## Notes

This project was supported by a generous grant from the Bill & Melinda Gates Foundation to the University of Oxford, for which we are very grateful. We thank the health authorities in Pelotas, Brazil; Beijing, China; Nagpur, India; Turin, Italy; Nairobi, Kenya; Muscat, Oman; Oxford, UK; and Seattle, USA; these health authorities facilitated the project by allowing participation of these study sites as collaborating centres. We also thank Philips Medical Systems, which provided the ultrasound equipment and technical assistance throughout the project; MedSciNet UK, for setting up the INTERGROWTH-21^st^ website and for the development, maintenance, and support of the online data management system; the parents and infants who participated in the studies; and the more than 200 members of the research teams who made the implementation of this project possible. The participating hospitals included: Brazil, Pelotas (Hospital Miguel Piltcher, Hospital São Francisco de Paula, Santa Casa de Misericórdia de Pelotas, and Hospital Escola da Universidade Federal de Pelotas); China, Beijing (Beijing Obstetrics & Gynecology Hospital, Shunyi Maternal & Child Health Centre, and Shunyi General Hospital); India, Nagpur (Ketkar Hospital, Avanti Institute of Cardiology, Avantika Hospital, Gurukrupa Maternity Hospital, Mulik Hospital & Research Centre, Nandlok Hospital, Om Women’s Hospital, Renuka Hospital & Maternity Home, Saboo Hospital, Brajmonhan Taori Memorial Hospital, and Somani nursing home); Kenya, Nairobi (Aga Khan University Hospital, MP Shah Hospital, and Avenue Hospital); Italy, Turin (Ospedale Infantile Regina Margherita Sant’ Anna and Azienda Ospedaliera Ordine Mauriziano); Oman, Muscat (Khoula Hospital, Royal Hospital, Wattayah Obstetrics & Gynaecology Poly Clinic, Wattayah Health Centre, Ruwi Health Centre, Al-Ghoubra Health Centre, and Al-Khuwair Health Centre); UK, Oxford (John Radcliffe Hospital); and USA, Seattle (University of Washington Hospital, Swedish Hospital, and Providence Everett Hospital). Full acknowledgment of all those who contributed to the development of the INTERGROWTH-21^st^ Project protocol appears at www.intergrowth21.org.uk. **Members of the International Fetal and Newborn Growth Consortium for the 21^st^ Century (INTERGROWTH-21^st^) and its committees** **Scientific advisory committee** M Katz (chair from January 2011), MK Bhan, C Garza, S Zaidi, A Langer, PM Rothwell (from February 2011), D Weatherall (chair until December 2010). **Steering committee** ZA Bhutta (chair), J Villar (principal investigator), S Kennedy (project director), DG Altman, FC Barros, E Bertino, F Burton, M Carvalho, L Cheikh Ismail, WC Chumlea, MG Gravett, YA Jaffer, A Lambert, P Lumbiganon, JA Noble, RY Pang, AT Papageorghiou, M Purwar, J Rivera, C Victora. **Executive committee** J Villar (chair), DG Altman, ZA Bhutta, L Cheikh Ismail, S Kennedy, A Lambert, JA Noble, AT Papageorghiou. **Project coordinating unit** J Villar (head), S Kennedy, L Cheikh Ismail, A Lambert, AT Papageorghiou, M Shorten, L Hoch (until May 2011), HE Knight (until August 2011), EO Ohuma (from September 2010), C Cosgrove (from July 2011), I Blakey (from March 2011). **Data analysis group** DG Altman (head), EO Ohuma, J Villar. **Data management group** DG Altman (head), F Roseman, N Kunnawar, SH Gu, JH Wang, MH Wu, M Domingues, P Gilli, L Juodvirsiene, L Hoch (until May 2011), N Musee (until June 2011), H Al-Jabri (until October 2010), S Waller (until June 2011), C Cosgrove (from July 2011), D Muninzwa (from October 2011), EO Ohuma (from September 2010), D Yellappan (from November 2010), A Carter (from July 2011), D Reade (from June 2012), R Miller (from June 2012). **Ultrasound group** AT Papageorghiou (head), LJ Salomon (senior external adviser), A Leston, A Mitidieri, F Al-Aamri, W Paulsene, J Sande, WKS Al-Zadjali, C Batiuk, S Bornemeier, M Carvalho, M Dighe, P Gaglioti, N Jacinta, S Jaiswal, JA Noble, K Oas, M Oberto, E Olearo, MG Owende, J Shah, S Sohoni, T Todros, M Venkataraman, S Vinayak, L Wang, D Wilson, QQ Wu, S Zaidi, Y Zhang, P Chamberlain (until September 2012), D Danelon (until July 2010), I Sarris (until June 2010), J Dhami (until July 2011), C Ioannou (until February 2012), CL Knight (from October 2010), R Napolitano (from July 2011), S Wanyonyi (from May 2012), C Pace (from January 2011), V Mkrtychyan (from June 2012). **Anthropometry group** L Cheikh Ismail (head), WC Chumlea (senior external adviser), F Al-Habsi, ZA Bhutta, A Carter, M Alija, JM Jimenez-Bustos, J Kizidio, F Puglia, N Kunnawar, H Liu, S Lloyd, D Mota, R Ochieng, C Rossi, M Sanchez Luna, YJ Shen, HE Knight (until August 2011), DA Rocco (from June 2012), IO Frederick (from June 2012). **Neonatal group** ZA Bhutta (head), E Albernaz, M Batra, BA Bhat, E Bertino, P Di Nicola, F Giuliani, I Rovelli, K McCormick, R Ochieng, RY Pang, V Paul, V Rajan, A Wilkinson, A Varalda (from September 2012). **Environmental health group** B Eskenazi (head), LA Corra, H Dolk, J Golding, A Matijasevich, T de Wet, JJ Zhang, A Bradman, D Finkton, O Burnham, F Farhi. **Participating countries and local investigators** Brazil: FC Barros (principal investigator), M Domingues, S Fonseca, A Leston, A Mitidieri, D Mota, IK Sclowitz, MF da Silveira. China: RY Pang (principal investigator), YP He, Y Pan, YJ Shen, MH Wu, QQ Wu, JH Wang, Y Yuan, Y Zhang. India: M Purwar (principal investigator), A Choudhary, S Choudhary, S Deshmukh, D Dongaonkar, M Ketkar, V Khedikar, N Kunnawar, C Mahorkar, I Mulik, K Saboo, C Shembekar, A Singh, V Taori, K Tayade, A Somani. Italy: E Bertino (principal investigator), P Di Nicola, M Frigerio, G Gilli, P Gilli, M Giolito, F Giuliani, M Oberto, L Occhi, C Rossi, I Rovelli, F Signorile, T Todros. Kenya: W Stones and M Carvalho (co-principal investigators), J Kizidio, R Ochieng, J Shah, J Sande, S Vinayak, N Musee (until June 2011), C Kisiang’ani (until July 2011), D Muninzwa (from August 2011). Oman: YA Jaffer (principal investigator), J Al-Abri, J Al-Abduwani, FM Al-Habsi, H Al-Lawatiya, B Al-Rashidiya, WKS Al-Zadjali, FR Juangco, M Venkataraman, H Al-Jabri (until October 2010), D Yellappan (from November 2010). UK: S Kennedy (principal investigator), L Cheikh Ismail, AT Papageorghiou, F Roseman, A Lambert, EO Ohuma, S Lloyd, R Napolitano (from July 2011), C Ioannou (until February 2012), I Sarris (until June 2010). USA: MG Gravett (principal investigator), C Batiuk, M Batra, S Bornemeier, M Dighe, K Oas, W Paulsene, D Wilson, IO Frederick, HF Andersen, SE Abbott, AA Carter, H Algren, DA Rocco, TK Sorensen, D Enquobahrie, S Waller (until June 2011).

Contributors: JV and SHK were responsible for conceiving the INTERGROWTH-21st Project. JV, SHK, DGA, and JAN prepared the original protocol with later input from ATP, LCI, and ZAB. JV, ATP, LCI, AL, and ZAB supervised and coordinated the project’s overall undertaking. EOO performed the statistical analysis in collaboration with DGA. EOO and DGA were responsible for data management and analysis in collaboration with JV and ATP. RP, EB, MC, YAJ, MFdS, MGG, and MP were collaborators and implemented the project in their respective countries. ATP, EOO, SHK, and JV wrote the paper in collaboration with CV, MGG, and JH, and with input from all coauthors. ZAB, SHK, and JV are joint senior authors. All coauthors read the report and made suggestions about its content. JV is guarantor.

Ethical approval: The INTERGROWTH-21^st^ Project was approved by the Oxfordshire research ethics committee C (reference 08/H0606/139), the research ethics committees of the individual participating institutions, and the corresponding regional health authorities where the project was implemented. All participants gave informed consent before taking part.

Data sharing: The relevant anonymised patient level data are available on reasonable request from the corresponding author.

The lead author affirms that the manuscript is an honest, accurate, and transparent account of the study being reported; that no important aspects of the study have been omitted; and that any discrepancies from the study as planned (and, if relevant, registered) have been explained.
