# Supplementary figures and images for: International standards for symphysis-fundal height based on serial measurements from the Fetal Growth Longitudinal Study of the INTERGROWTH-21st Project: prospective cohort study in eight countries
Source: BMJ. 2016 Nov 7;355:i5662. doi: 10.1136/bmj.i5662 (PMC5098415; doi:10.1136/bmj.i5662)

# International Standards for Symphysis-Fundal Height

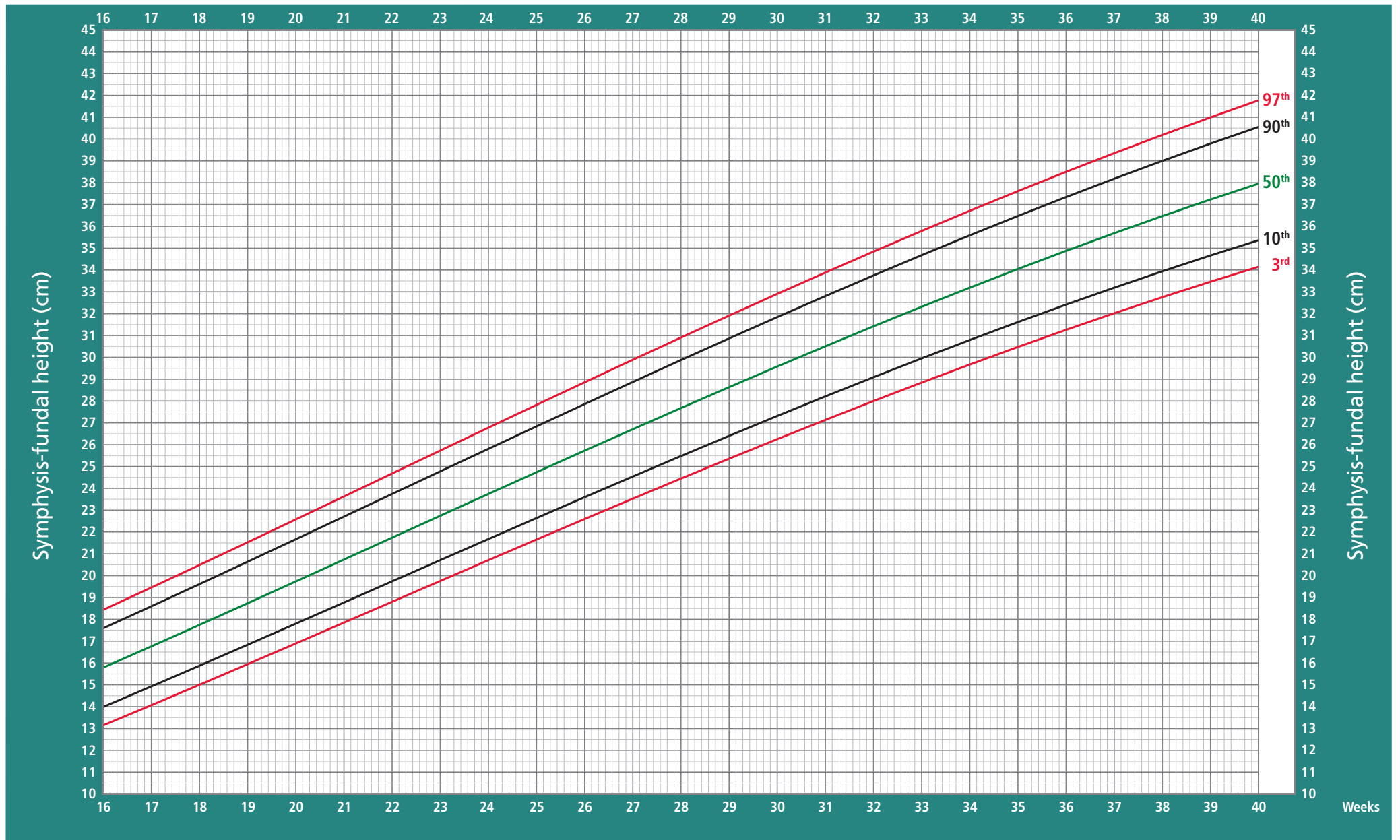

Supplement: Supplementary file 1 — Supplementary appendix: International Standards for Symphysis-Fundal Height [file papa034430.ww_default.pdf]
